# Using AI and BES/MFC to decrease the prediction time of BOD_5_ measurement

**DOI:** 10.1007/s10661-023-11576-0

**Published:** 2023-08-05

**Authors:** Ivan Medvedev, Mariya Kornaukhova, Christoforos Galazis, Bálint Lóránt, Gábor Márk Tardy, Alexander Losev, Igor Goryanin

**Affiliations:** 1 Volgograd State University, Volgograd, Russia; 2 Imperial College London, London, UK; 3 Budapest University of Technology and Economics, Budapest, Hungary; 4 University of Edinburgh, Edinburgh, UK; 5 Okinawa Institute Science and Technology, Okinawa, Japan

**Keywords:** Neural network, Biochemical Oxygen demand, Biosensor, Microbial fuel cell

## Abstract

Biochemical oxygen demand (BOD) is one of the most important water/wastewater quality parameters. BOD_5_ is the amount of oxygen consumed in 5 days by microorganisms that oxidize biodegradable organic materials in an aerobic biochemical manner. The primary objective of this research is to apply microbial fuel cells (MFCs) to reduce the time requirement of BOD_5_ measurements. An artificial neural network (ANN) has been created, and the predictions we obtained for BOD_5_ measurements were carried out within 6–24 h with an average error of 7%. The outcomes demonstrated the viability of our AI MFC/BES BOD_5_ sensor in real-life scenarios.

## Introduction

A microbial fuel cell (MFC) is a device that converts the energy of chemical bonds of organic substances into an electric current by the metabolism of specific (so-called exoelectrogenic) bacteria. In recent years, extensive research has been conducted to elaborate MFC-based biosensors to evaluate the quality of wastewater. A dual-chamber MFC is made up of an ion-selective membrane that can only allow protons to flow through, as well as anode and cathode chambers, while a single-chamber MFC consists of an anode chamber and an air cathode. Electroactive bacteria form a biofilm over the anode electrode, generating electrons during the oxidation of organic materials which they transport to the electrode surface. As a result, the biodegradable organic content of the water can be calculated from the electric output. Yang et al. (2015) and Lóránt et al. (2019) claim that this technology can detect and even quantify dangerous toxic or organic substances. Thanks to electrogenic bacteria that can transform the chemical energy stored in organic material into electrical energy, MFCs may be used as an alternative technology to determine the extent of water contamination. MFCs are energy-efficient devices that can clean water, provide power sufficient to run low-energy devices, monitor water quality, and find dangerous compounds all at on (Rabaey & Verstraete, 2005).

A previous study (Tardy et al., 2021) proposed a method for using MFCs as biosensors to measure 5-day biochemical oxygen demand (BOD_5_). The specific prices of the currently high-cost materials (membrane, cathode catalysts) will expectedly highly decrease due to the new materials and the production of these materials in higher volumes. In our technology, an alternative catalyst material has been applied at a considerably lower price than the platinum-based ones (Patent number: US20190393532A1), and cathode patent (US20100297477A1) also separator patent exchange membrane was much cheaper than Nafion® and more versatile (WO/2019/160046). It is true that currently several different MFC designs and different membranes are applied by different research groups worldwide, and indeed, these technologies are in the development stage. However, among several other types of research (see e.g., 10.3390/bios9030092; 10.1021/acssensors.0c01299?ref=pdf), we aim to find the MFC-based technology which is competitive enough to be standardized. Having it standardized can be a good alternative to the current BOD5 standard.

BOD_5_ is one of the most important parameters to assess water pollution levels by biodegradable organic substances. Environmental agencies use it to monitor wastewater treatment plants and natural water resources. Ongoing experiments with microbial fuel cells (MFCs) as biosensors have reduced the time required to obtain the initial data needed to predict BOD_5_ in wastewater (Tardy et al., 2021). The method is based on the correlation between the total amount of generated electricity and the BOD_5_ of the sample. Compared to the conventional respirometric method with a fix 5-day long measurement, the prediction time was reduced to 1–4 days dependent on the composition of the investigated sample (Tardy et al., 2021).

Mathematical models can be used to model the processes occurring in the MFC, for example, work (Pinto et al., 2010), in which a dynamic model of a single-chamber MFC was developed based on an ordinary differential equation, which reflects the dynamics of the anode chamber, considering two populations of microorganisms. But for the application of mathematical models, as a rule, deep knowledge of MFC systems is required. Some of the main limitations of the application of mathematical models to MFCs are, for example, the low predictability of their operation and the influence of certain environmental conditions on the efficiency of MFCs (Picioreanu et al., 2010).

Alternative modeling methods are artificial intelligence, in particular ANN. As in other areas of research, the use of ANNs for MFС data has attracted the attention of many researchers. For example, in Tsompanas et al. (2019), ANNs were used to model the polarization curves of various ceramic MFC settings. And in Ismail et al. (2019), a three-layer ANN was used to predict the power generation of two-chamber MFCs continuously fed with domestic wastewater enriched with giant reed as a new energy source. Machine learning approaches such as the relevant vector method (RVM) and accelerated genetic algorithm (AGA) global search algorithm were proposed in a study (Fang et al., 2013) to optimize the operation of MFC with multiple variables. The work (Garg et al., 2014) compares artificial intelligence methods such as ANN, MGGP, and SVR for MFC performance modeling. And a study (Tsompanas et al., 2021) examines the application of NARX networks to predict the electrical output of an MFC given its previous outputs.

Due to the fact that the data obtained in the study (Tardy et al., 2021) have not yet been evaluated in detail, the knowledge to use standard mathematical methods for these data has not yet been given in the literature. Because of this, it was concluded that it is possible to use ANN. Therefore, the use of ANNs in this problem is primarily because their use does not require deep knowledge about MFCs and their operation, in contrast to standard methods of mathematical modeling.

An artificial neural network (ANN) is one of the well-known predictive methods used to find a solution when other statistical methods are not applicable. The advantages of using ANNs are the ability to learn from training data and to predict non-linear data, making ANNs a widely used statistical tool. In this work, fully connected multilayer neural networks–multilayer perceptron (MLP) were used. MLPs are classical feedforward neural networks that are used in both regression and classification problems (Pal & Mitra, 1992). MLPs are widely used in various fields such as remote sensing (Zhang et al., 2018) and engineering (Yilmaz & Kaynar, 2011) or plant sciences (Yoosefzadeh-Najafabadi et al., 2021) and environmental sciences (Wang & Gao, 2018).

Two approaches were considered, in the first the ANN directly predicted one BOD_5_ value from the raw electrical parameters. In the second, the ANN used electrical data obtained in MFCs, and the total charge was calculated. Furthermore, based on the linear dependence of BOD_5_ and the charge, the predicted values of BOD_5_ were obtained and compared with BOD_5_ data measured with the standard respirometric method.

## Material and methods

### Data set

The dataset was obtained using MFCs described by Tardy et al. (2021). The main purpose of a microbial fuel cell biosensor is to convert the chemical energy of biodegradable organic substances to electrical energy by the metabolic processes of exoelectrogenic bacteria that can transport the generated electrons outside the cell. The amount of generated electricity (voltage, current) is recorded. The study is based on the conclusion about the linear dependence of the biochemical oxygen demand (BOD_5_) and the charge accumulated during the biodegradation in the MFC (Tardy et al., 2021).

Two types of wastewater were used as samples for MFC: domestic and brewery wastewater. Three identical air cathode MFCs were operated in parallel, with a 230-ml internal volume each. The volume of the injected substrate was 60 ml. In some cases, the wastewater samples were diluted to cover a wider range of BOD_5_. The external resistance was set equal to 100 ohms (Tardy et al., 2021).

**Fig. 1:**
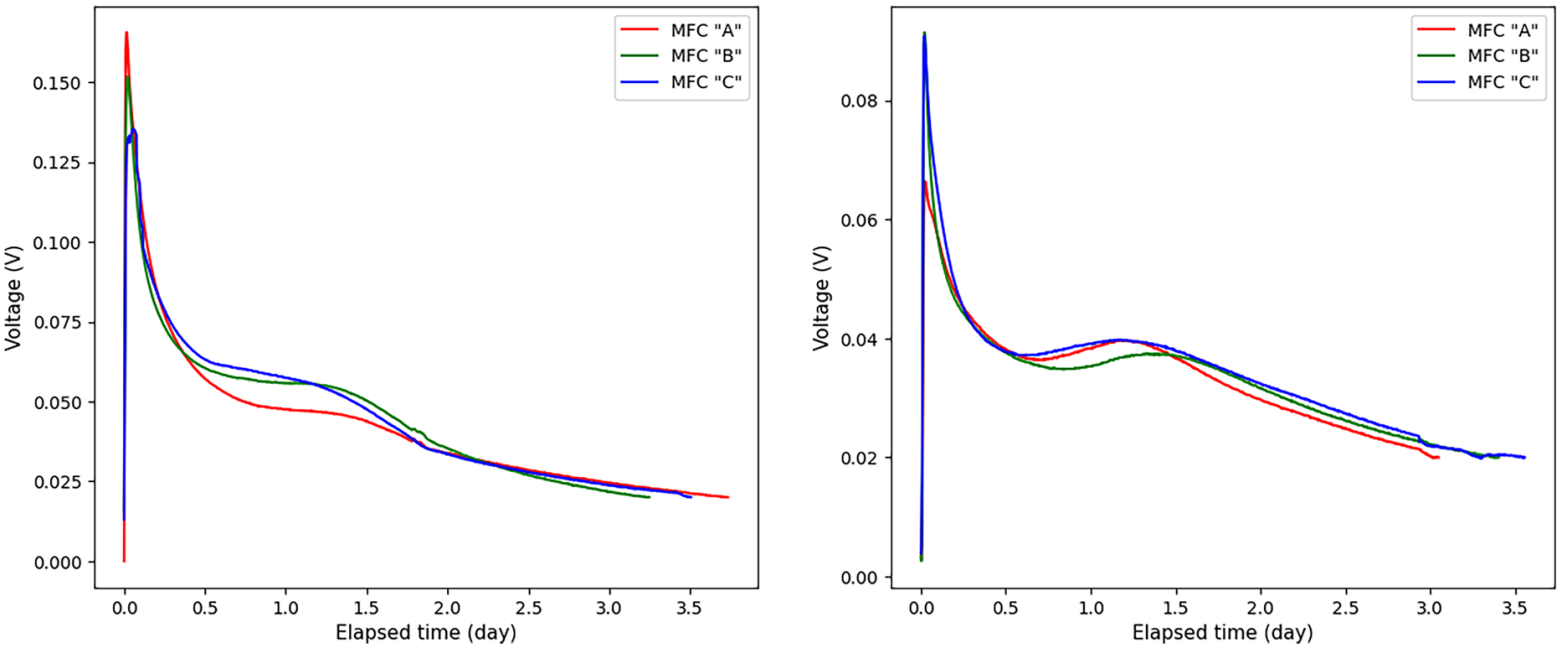
Several typical examples of voltage variation over time, when domestic wastewater was used as a sample for MFC

**Fig. 2:**
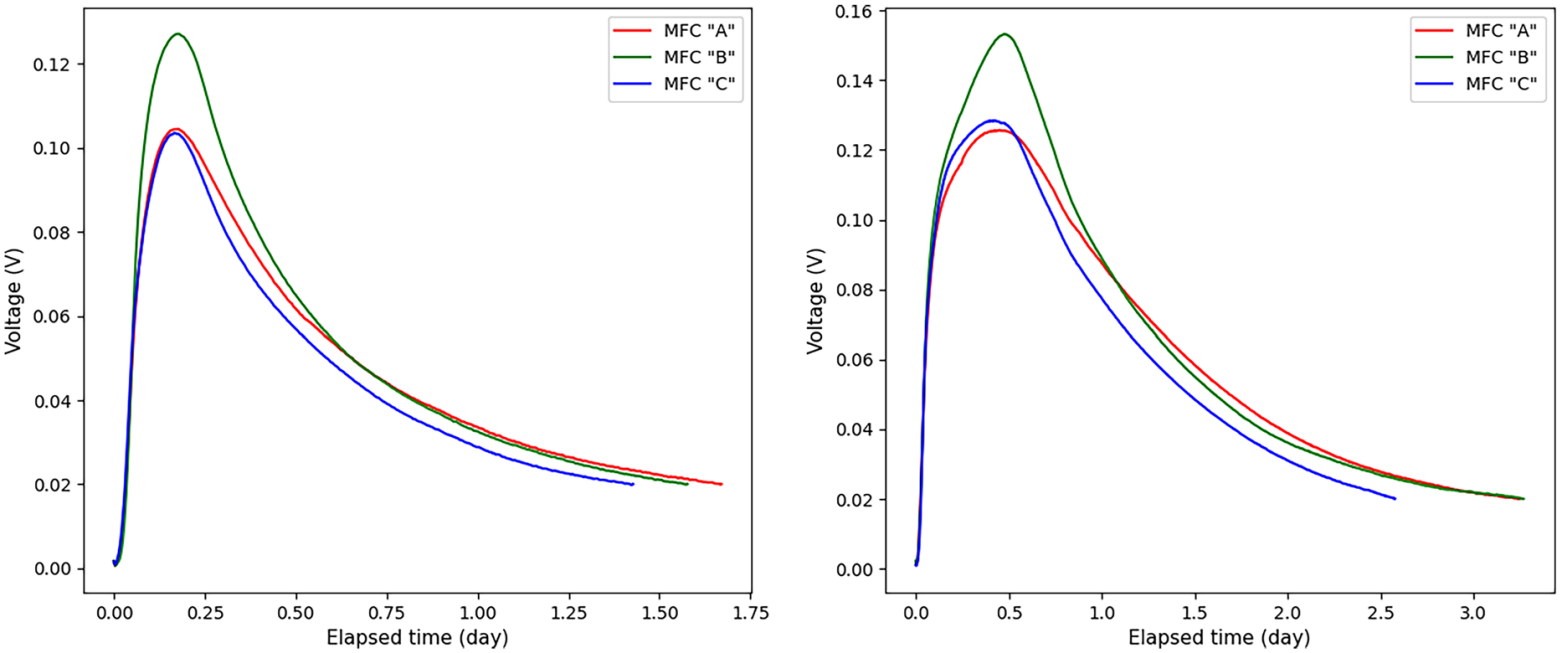
Several typical examples of voltage variation over time, are when brewery wastewater was used as samples for MFC

During the measurements, the voltage was recorded by the data acquisition device (Graphtec midi logger GL840) every 5 min. It should be noted that in the initial period of each experiment, the voltage increased rapidly and reached its maximum value as a result of the rapid biodegradation of a readily biodegradable organic fraction of the wastewater. Having the readily biodegradable substrates consumed, the voltage value began to drop. When the voltage dropped below 0.02 V, the substrates were considered to be depleted and the measurements were terminated. Figure 1 shows several examples of voltage measurement plots when domestic wastewater was used. Examples of voltage graphs for water samples from breweries are shown in Fig. 2.

In this work, a set of 56 voltage measurements was used. The longest experiment was 7550 min, so to equalize the dimension of all experiments, the missing values of other experiments were filled with zeros up to 7550 min. Since the voltage values in each experiment were recorded every 5 min, then 1511 discrete voltage values corresponded to 7550 min. Thus, the voltage dataset was presented as a matrix of 56 columns and 1511 rows. It is worth noting that 289 discrete voltage values corresponded to 24 h of measurement, we also note that 16, 12, 8, 6, and 2 h of measurement corresponded to 193, 145, 97, 73, and 25 discrete voltage values. In addition, the BOD_5_ concentrations in mg corresponding to each experiment were reported in the data set. The BOD_5_ data is represented as a vector of 56 values (*y*_1_, *y*_2_, …, *y*_56_), where each *y*_*j*_ the value corresponds to the BOD_5_ value in the *j*th experiment. The minimum BOD_5_ content of the 60-ml samples was 4.13 mg and the maximum content was 46.84 mg, corresponding to a wide BOD_5_ concentration range from ~69 to 781 mg/L.

### Neural networks

The purpose of this study was to develop artificial neural network models for the prediction of BOD_5_. We used fully connected multilayer neural networks (multilayer perceptron (MLP))—a classical feedforward neural network, which consists of an input layer, an output layer, and intermediate layers (hidden layers), each of which consists of several neurons. The value in each of the neurons is the value of the weighted sum of all neuron values from the previous layer, converted through the activation function, plus the bias coefficient. MLP is effective in regression problems, for example (Wang & Gao, 2018), MLP gave good results in predicting the water content of biodiesel and diesel blends in terms of temperature and composition, and for predicting gas density (Sedaghat & Kiomarsiyan, 2019).

As described in the introduction, two approaches were considered. The BOD_5_ direct prediction approach was that the ANNs predict one value—BOD_5_ in each experiment. The approach of indirect prediction of BOD_5_ consisted of the ANNs output voltage values, from which BOD_5_ values were subsequently calculated. When implementing both approaches, the voltage values obtained for 24, 16, 12, 8, 6, and 2 h of measurements were used as input data for the ANN, i.e., it was required to develop 12 ANN models in total, six ANNs for each approach.

The input data set was represented by 56 vectors (*x*_1*j*_, *x*_2*j*_, …, ${x}_{n_1j}$), where *x*_ij_ is the voltage value at the *i*th moment in time and *j*th experiment; *n*_1_ = 289, 193, 145, 97, 73, 25, which corresponds to the length of the voltage vectors for the first 24, 16, 12, 8, 6, and 2 h of measurements, respectively; *j* = 1, …, 56. That is, the number of neurons in the input layers in the implementation of both approaches was 289, 193, 145, 97, 73, and 25. In each experiment, the final voltage value was reached at different times, for example, one experiment ran for two days and another for 5 days, but the output of the neural network requires these values to be of the same time. Therefore, the voltage measurement was complimented with zero values to generate 5-day time sequences.

Three hidden layers were used for the neural networks, for each of which the Rectified linear unit (ReLU) activation function was used. In addition, after each hidden layer, a thinning (dropout) method was used to reduce overfitting. This method consists of the elimination of a certain percentage of random neurons at different iterations during neural network training (Srivastava et al., 2014). Then, the output layer was followed, in which the dimensions differed depending on which of the two approaches was used. So, when implementing the BOD_5_ direct prediction, the number of neurons in the output layer was one for all six ANNs, since only the BOD_5_ value was predicted. The set of output data for direct prediction can be represented as a vector of 56 values (*y*_1_, *y*_2_, …, *y*_56_), where each value of *y*_*j*_ corresponds to the BOD_5_ value in the *j*th experiment.

When BOD_5_ the indirect prediction was implemented, the output set was represented by 56 vectors (*y*_1*j*_, *y*_2*j*_, …, ${y}_{n_2j}$), where *y*_*ij*_ is the voltage value at the *i*th time point in the *j*th experiment; *n*_2_ = 1486, 1438, 1414, 1366, 1318, 1222, which corresponds to the length of the voltage vectors obtained after 2, 6, 8, 12, 16, and 24 h of measurements, respectively; *j* = 1, …, 56. Therefore, the number of neurons in the output layer for the indirect prediction was 1486, 1438, 1414, 1366, 1318, and 1222 respectively, which matches the voltage vectors for each time measurement.

ANN parameters were selected in the process of cross-validation for 5 folds in order to minimize errors (1) and (2) on the validation blocks. Initially, three-layer ANNs with 128 neurons on each of the hidden layers were created as initial models. After cross-validation, the number of neurons in the hidden layers changed (in particular, options from 128, 64, and 96 neurons were selected on each of the hidden layers). In addition, dropout layers were added (on the dropout layers, such variants of neuron exclusion percentages as 10%, 20%, 25%, 30%, 50%) were selected.

The article shows the results without preliminary normalization to the data, but we also conducted computational experiments with normalization. In particular, we performed normalization according to the mini-max formula, which is widely used in solving machine learning problems. The maximum and minimum voltage values were chosen expertly, since during the input time the voltage could not always reach its peak or minimum value. As the maximum value for each experiment, we used the value of the voltage at the peak or the value of the voltage close to the peak (in case the voltage did not reach the peak during the input time), and as the minimum value, we took 0.02 or less voltage value and obtained comparable results. with those that we present in the article. But for a shorter time (less than 12 hours), the results with normalization were worse, since it was difficult to expertly evaluate the maximum and minimum voltages from the available input data.

The parameters used for neural networks in the BOD_5_ direct prediction approach is shown in Table 1, and the indirect prediction approach in Table 2.

**Table 1:** Neural network parameters for the direct prediction approach

Input	Dense 1	Dropout1	Dense 2	Dropout2	Dense 3	Dropout3	Output
25	128	30%	128	30%	64	50%	1
73	128	10%	128	50%	64	50%	1
97	128	10%	64	30%	64	50%	1
145	96	10%	96	10%	64	40%	1
193	128	10%	128	30%	128	30%	1
289	128	10%	128	10%	64	30%	1

**Table 2:** Neural network parameters for the indirect prediction approach

Input	Dense 1	Dropout1	Dense 2	Dropout2	Dense 3	Dropout3	Output
25	128	30%	128	30%	64	50%	1486
73	128	20%	64	50%	64	50%	1438
97	128	25%	128	50%	64	50%	1414
145	128	10%	64	30%	64	50%	1366
193	128	10%	64	10%	64	50%	1318
289	128	10%	128	20%	64	50%	1222

**Fig. 3:**
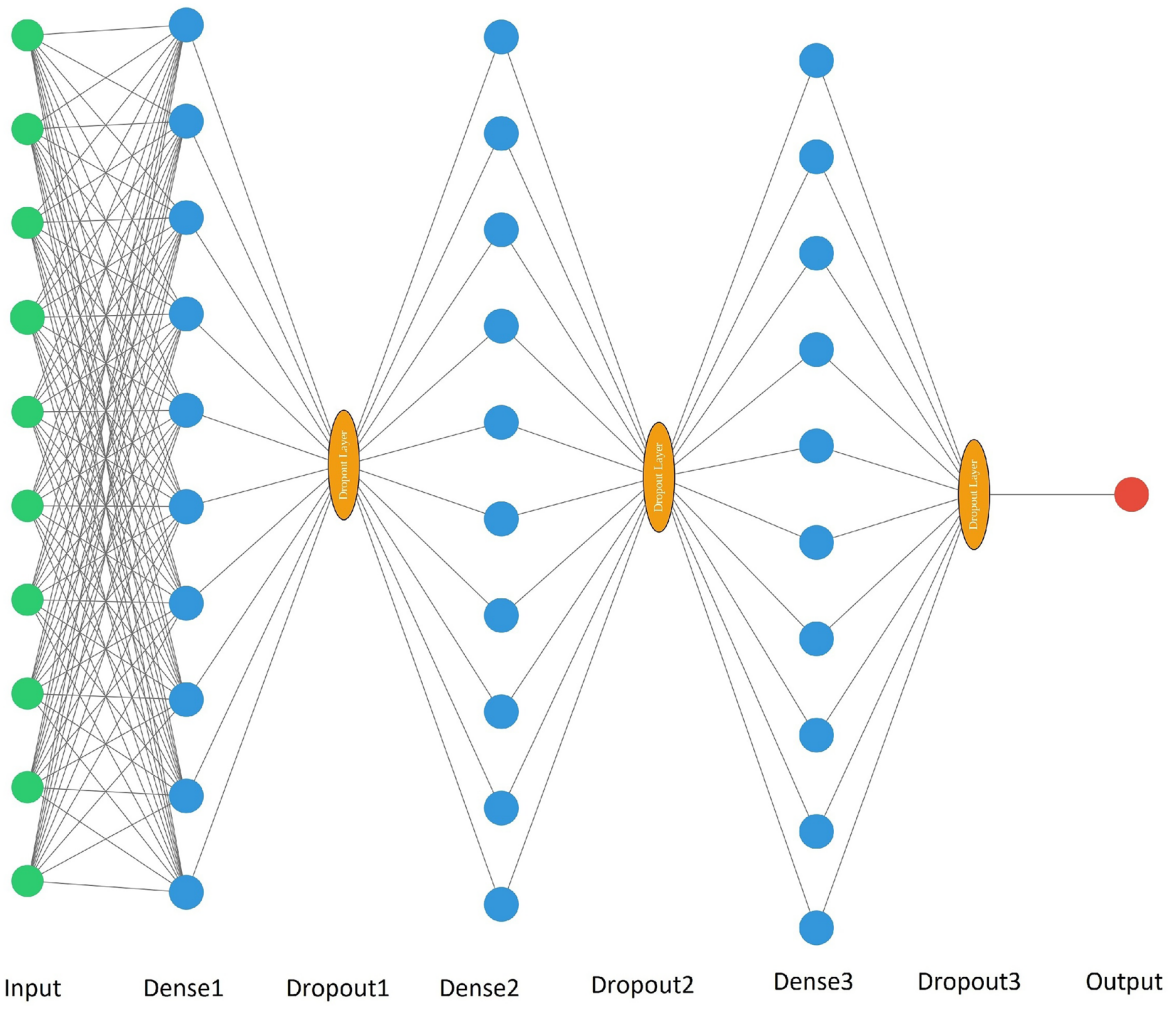
Schematic drawing of neural network architectures for a direct prediction approach. Where the input, dense, dropout, and output values correspond to the values from Table 1. After the hidden layers, the neuron values pass through the Relu activation function

**Fig. 4:**
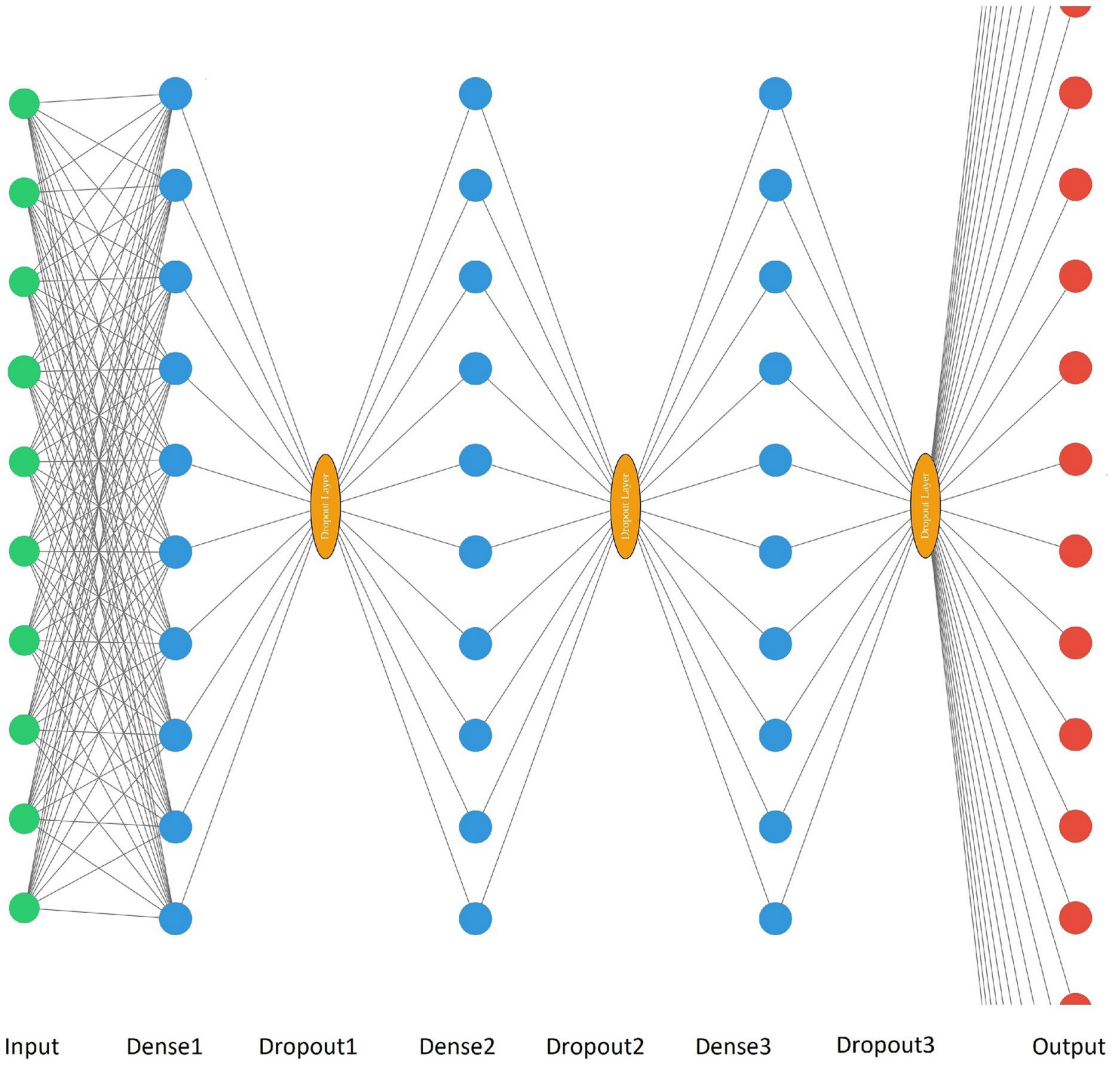
Schematic drawing of neural network architectures for an indirect prediction approach. Where the input, dense, dropout, and output values correspond to the values from Table 2. After the hidden layers, the neuron values pass through the Relu activation function

Schematic drawings of neural network architectures are shown in Figs. 3 and 4 for the direct and indirect prediction approaches, respectively. The parameters used for neural networks in the BOD_5_ direct prediction approach is shown in Table 1, and the indirect prediction approach in Table 2.

As described earlier, a dataset of 56 experiments was used in this work. Of these, 16 experiments (8 experiments with domestic wastewater and 8 experiments with wastewater from breweries) were used as the test set for the final evaluation of the models once model tuning and training were completed. With the remaining 40 experiments, the *K*-fold cross-validation (*K*=5) method was applied to assess the quality of the ANNs during parameter selection. The data set of 40 experiments were divided into 5 blocks, with each block clustered into 8 experiments. In the first stage, the first block (20% of the data) was used as a validation block, and the remaining 4 blocks (80% of the data) were used as training. In the next stage, the second block (20% of the data) was used as validation and the remaining blocks (80% of the data) as training data. And so on, until each block of 20% has been used in the validation.

According to the obtained five estimates, the average value of the loss function was calculated. The root means square error (MSE) was defined as the loss function to be minimized during training. Moreover, when implementing the BOD_5_ direct prediction approach, the MSE of the predicted BOD_5_ values relative to the actual BOD_5_ values were minimized. The formula for the direct MSE is (1):

1 $${\textrm{MSE}}_1=\frac{1}{n}\sum\limits_{i=1}^n{\left({y}_{\textrm{i}}-\overline{y_{\textrm{i}}}\right)}^2,$$

where *n* is the number of ANN output values (in all cases equal to 1) multiplied by the number of experiments in the validation set (there were 8 experiments in each of the 5 validation blocks); *y*_i_—real values of BOD_5_; $\overline{y_{\textrm{i}}}$—predicted values (the value of the weighted sum of all neuron values from the previous layer plus the bias factor converted through the activation function) BOD_5_.

And when implementing the BOD_5_ indirect prediction approach, the MSE of the predicted voltage values relative to the real ones was minimized. The formula for the indirect MSE is (2):

2 $${\textrm{MSE}}_2=\frac{1}{n\bullet {n}_2}\sum\limits_{j=1}^n\sum\limits_{i=1}^{n_2}{\left({y}_{\textrm{ij}}-\overline{y_{\textrm{ij}}}\right)}^2,$$

where *n* is the number of experiments in the validation set (there were 8 experiments in each of the 5 validation blocks); ); *n*_2_ = 1486, 1438, 1414, 1366, 1318, 1222, which corresponds to the length of the voltage vectors obtained after 2, 6, 8, 12, 16, and 24 h of measurements, respectively; y_ij_—real voltage values at the *i*th moment of time in the *j*th experiment; $\overline{y_{\textrm{ij}}}$—predicted stress values at the ith time point in the *j*th experiment.

To minimize the loss function during training, the Adam optimizer (adaptive moment) was used in this work. For the BOD_5_ direct prediction approach, the learning rate was 0.001, the rest of the parameters of the Adam method were left at the default settings for the Keras library.

For the BOD_5_ indirect prediction approach, the learning rate was 0.0001, the rest of the parameters of the Adam method were left at the default settings for the Keras library. Adam is an efficient stochastic optimization method that combines the benefits of methods such as AdaGrad and RMSProp (Kingma & Ba, 2015).

The calculations were carried out using Colab notebooks, which allow you to execute code on Google cloud servers. This means that it is possible to use Google hardware, including GPUs and TPUs, regardless of the power of the machine used by the developer, which is a significant advantage of this environment over others.

After the final selection of all parameters, such as the number of neurons in the layers and thinning percentages, the number of epochs for each of the 12 ANNs were selected, at which the average MSE over 5 validation blocks was minimal. These epochs for 12 ANNs were used to train the final ANNs on a sample of 40 experiments and tested on a leave-out sample of 16 experiments. Note that the errors of the neural network given in this paper are given taking into account the error in the operation of the microbial fuel cell. Namely, the error in detecting BOD_5_ using MFC gives an error within 10%, specifically for the test sample used, the average relative error in detecting BOD_5_ by real MFC voltage is approximately 7%. Thus, the neural network cannot improve the results of the MFC itself, since it is trained directly on the data of this device.

## Results and discussion

### Results of direct prediction of BOD_5_ using ANN

Six ANN models were developed for the BOD_5_ direct prediction approach. After training 40 sets in *K*-fold cross-validation, a set of weights were stored that resulted in a minimum loss value. These weights were applied for inference.

To compare actual and predicted BOD_5_ values, the mean absolute percentage error (MAPE) was used (3):

3 $$\textrm{MAPE}=\frac{1}{m}{\sum}_{i=1}^m\frac{\left|{y}_{\textrm{i}}-\overline{y_{\textrm{i}}}\right|}{\left|{y}_{\textrm{i}}\right|}100\%$$

and the maximum absolute error (MAX) (4):

4 $$\operatorname{MAX}=\max \left|{y}_{\textrm{i}}-\overline{y_{\textrm{i}}}\right|,\kern0.5em i=1,\dots, m,$$

where *m* = 16 when assessing BOD_5_ on the test set (because there were 16 experiments in the test set) and *m* = 8 when assessing BOD_5_ on each of the validation blocks; *y*_*i*_ – real values of BOD_5_; $\overline{y_{\textrm{i}}}$—predicted BOD_5_ values.

In addition to the errors described above, to estimate the predicted values of BOD_5_ relative to the actual values of BOD_5_, the coefficient of determination was used (*R*^2^) (5):

5 $${R}^2=1-\frac{\sum_{i=1}^m{\left(\overline{y_{\textrm{i}}}-{y}_{\textrm{i}}\right)}^2}{\sum_{i=1}^m{\left({y}_{\textrm{i}}-{\hat{y}}_{\textrm{i}}\right)}^2},$$

where ${\hat{y}}_{\textrm{i}}$ are the average overall real values of *y*_*i*_. The larger the value of *R*^2^ (close to 1), the better the accuracy of the linear relationship between the actual and predicted results.

The errors of the predicted BOD_5_ compared to the actual values of BOD_5_ for *K*-fold cross-validation are shown in Table 3.

**Table 3:** Mean errors over 5 validation blocks

Input	MAPE	MAX	*R*^2^
2 hours	39.63%	15.79 mg	0.208
6 hours	23.39%	9.56 mg	0.65
8 hours	20.06%	10.43 mg	0.73
12 hours	16.63%	9.22 mg	0.745
16 hours	12.4%	5.41 mg	0.845
24 hours	8.14%	3.39 mg	0.938

**Fig. 5:**
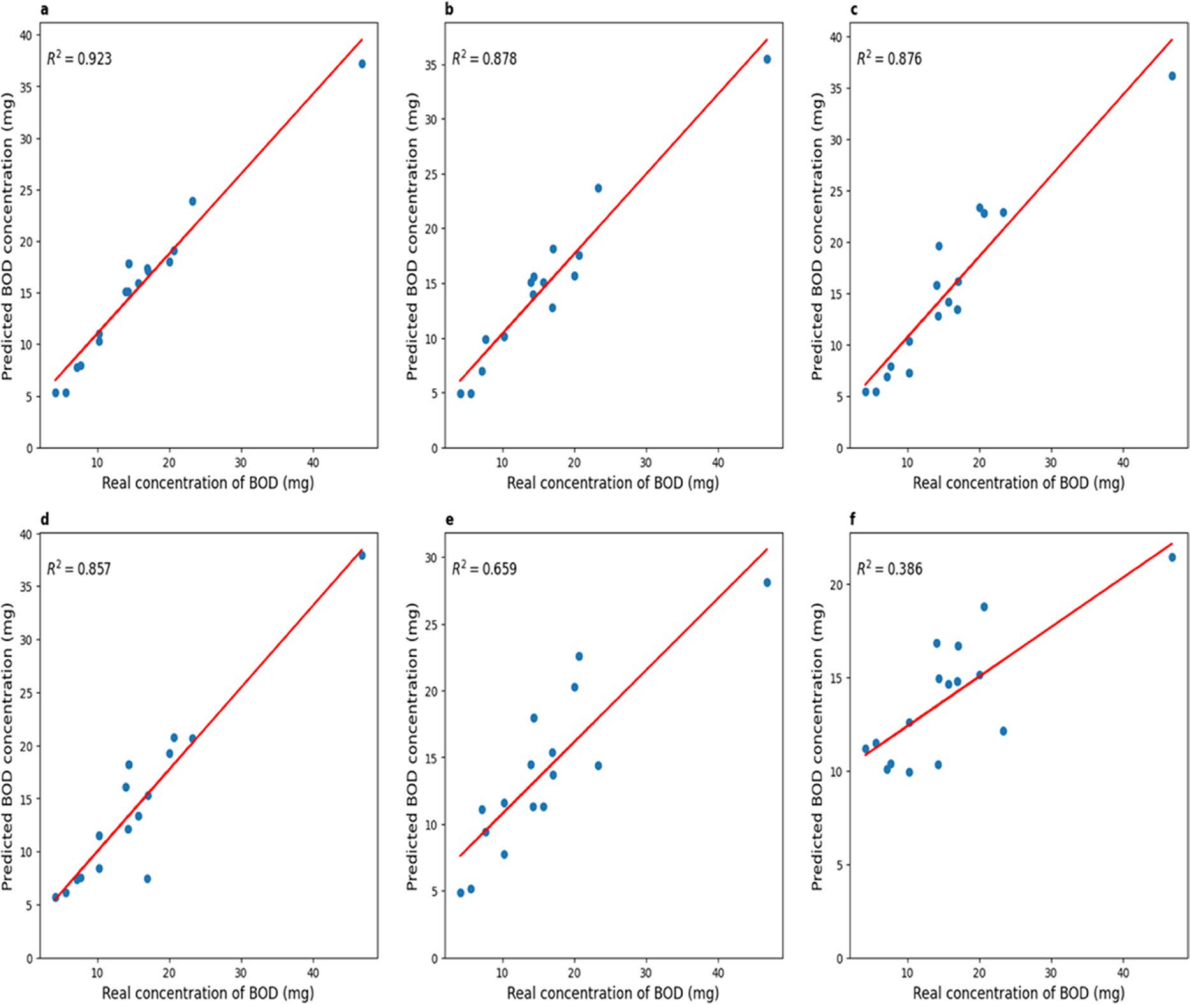
Plots comparing actual and predicted BOD_5_ values (for the direct prediction approach). **a** When using 24-h voltage data; **b.** for 16 h; **c** 12 h before; **d**. 8 h; **e** 6 h; **f** for 2 h

Next, the models were trained on 40 sets and tested on a delayed sample of 16 experiments. The results are shown in Table 4. Graphs of comparison of real and predicted BOD_5_ values are shown in Fig. 5.

**Table 4:** Model errors on delayed set

Input	MAPE	MAX	*R*^2^
2 hours	36.6%	25.36 mg	0.385
6 hours	21.02%	18.74 mg	0.659
8 hours	15.76%	9.46 mg	0.857
12 hours	13.59%	10.62 mg	0.875
16 hours	11.23%	11.33 mg	0.877
24 hours	8.72%	9.61 mg	0.923

It can be seen that the results on the test set do not differ considerably from the results obtained during cross-validation for 5 blocks. The models were able to predict the correct values, corresponding to the expected ones, when the voltage values for 24, 16, and 12 h were applied to the input (determination coefficient on the delayed sample: 0.923, 0.877, 0.875).

In total, there were 10 different types of wastewater with pollution and six types of wastewater without pollution in the test sample; therefore, indicators of specificity, sensitivity, and accuracy were also calculated to evaluate the neural network. Sensitivity was calculated as the ratio of the number of polluted waters correctly identified by the neural network to the true number of polluted waters in the test. Specificity was calculated as the number of unpolluted waters detected by the neural network to the true number of unpolluted waters in the test. Thus, the sensitivity was 1 when the input data was 24 h of measurements, 0.9 for the cases of 16 and 12 h, and 0.8 for the remaining cases (8, 6, and 2 h). The specificity was 1 for 24, 12, and 8 h of measurements, 0.83 for 16 h, and 0.67 for the 6 and 2-h cases. In addition to specificity and sensitivity, accuracy was also calculated as the ratio of the number of correctly guessed water states (contamination or not) to the amount of data in the test set (16 experiments). The accuracy was 1 for the entry at 24 h of measurement, 0.975 for 12 h, 0.875 for the entry at 16 and 8 h, and 0.75 for the entry at 6 and 2 h.

### Results of indirect *BOD*_5_ prediction using ANN

The indirect prediction approach of BOD_5_ using ANN was that, knowing the voltage and external resistance, which, as already described earlier, was set to 100 Ohm to accelerate the biodegradation process, the current strength can be obtained according to Ohm’s law. By numerically integrating the current over time, the total charge can be calculated as shown in the formula below (6):

6 $$Q=\int_{t_s}^{t_e}I\ \textrm{dt},$$

where *Q* is the total charge (*C*), *I* is the current in the external circuit (A), *t*_*s*_ (s) is the starting time of the experiment, *t*_*e*_ (s) is the end time of the measurement. Due to the linear relationship between BOD_5_ and charge, the resulting total charge can be used to estimate BOD_5_.

When implementing this approach, six ANN models were developed that predict voltage values after a certain measurement time. After selecting epochs that give the minimum mean square error over 5 validation blocks, ANNs were trained for 40 experiments with voltage measurement and tested on 16 experiments from the test set.

**Fig. 6:**
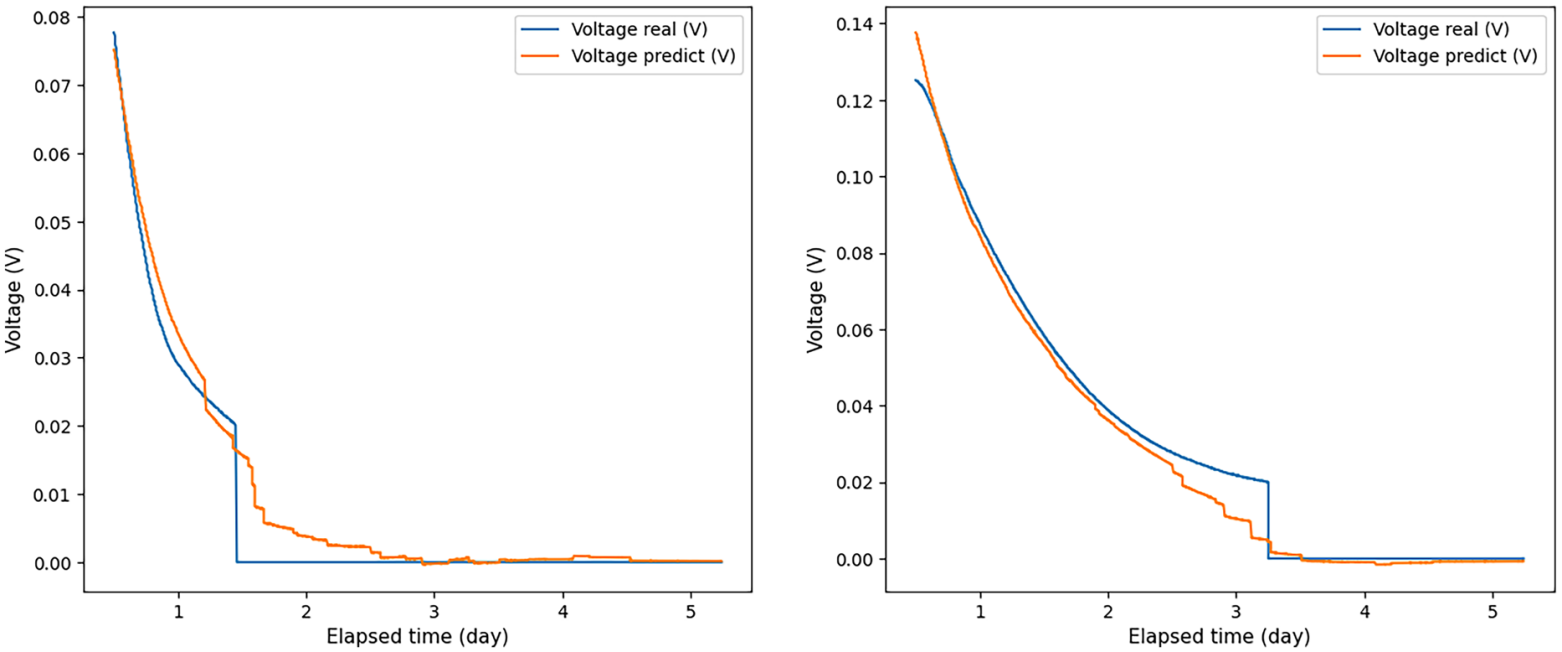
A few examples of the ratio of real to predicted voltage when voltage values measured over 12 h were used as input to the ANN. Blue graph—real voltage data; orange—obtained using a neural network

The ANN was able to predict the correct values corresponding to those expected when the input voltage values were applied for 24, 16, and 12 h and in some cases even 8 and 6 h. For example, some graphs of the predicted and experimental voltage values, when voltage values obtained for 12 h were applied to the ANN input, are shown in Fig. 6.

**Fig. 7:**
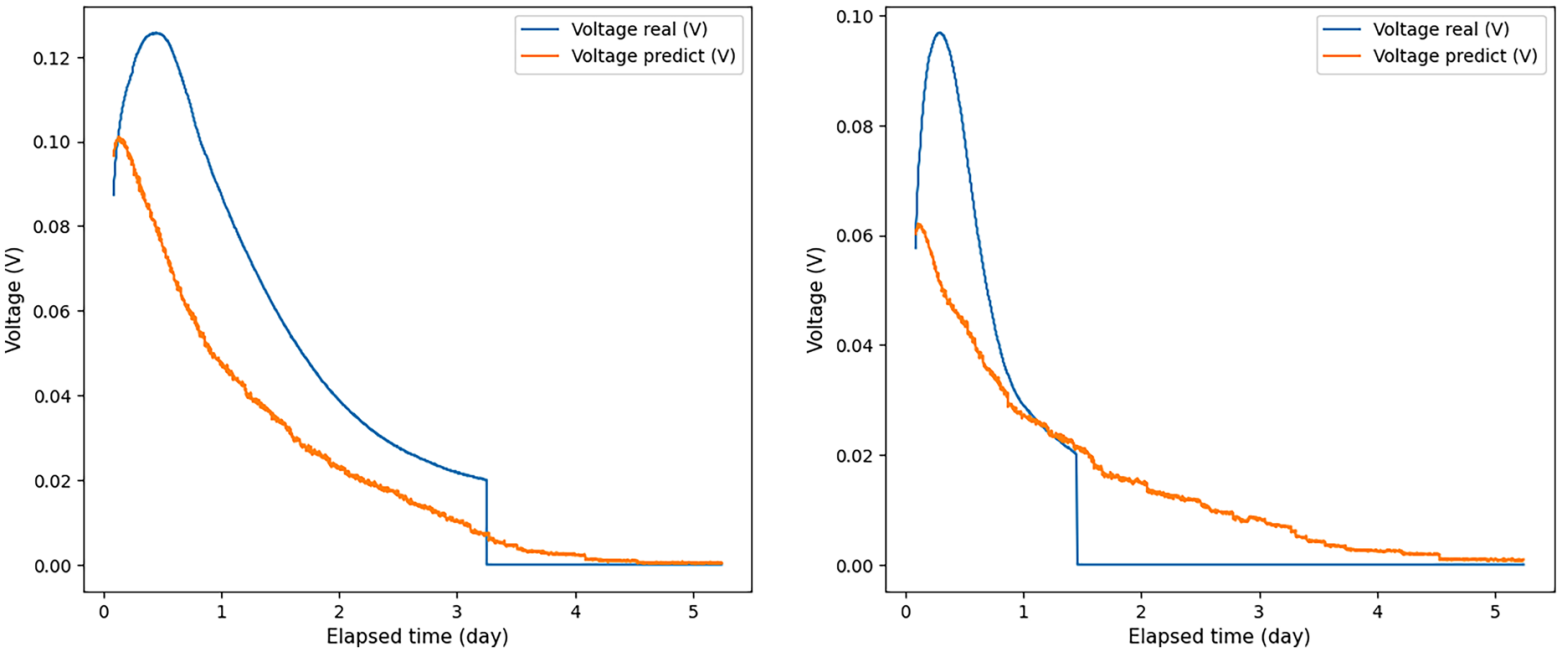
A few examples of the ratio of real voltage to predicted voltage, when voltage values measured over 2 h were used as input data for the ANN. Blue graph—real voltage data; orange—obtained using a neural network

As a rule, in experiments in which the voltage did not peak and did not begin to decrease after the input time allowed, the results were lower compared to those experiments in which the voltage peak was reached before the time of the input data measurement. Therefore, using voltage values obtained for 6 and 2 h as input data, the measurement results deteriorated significantly in comparison with other cases (see Fig. 7, which shows the ratio of the real and predicted voltage graphs, when voltage values were applied to the ANN input received within 2 h). The voltage did not peak and did not begin to decrease by this time.

**Fig. 8:**
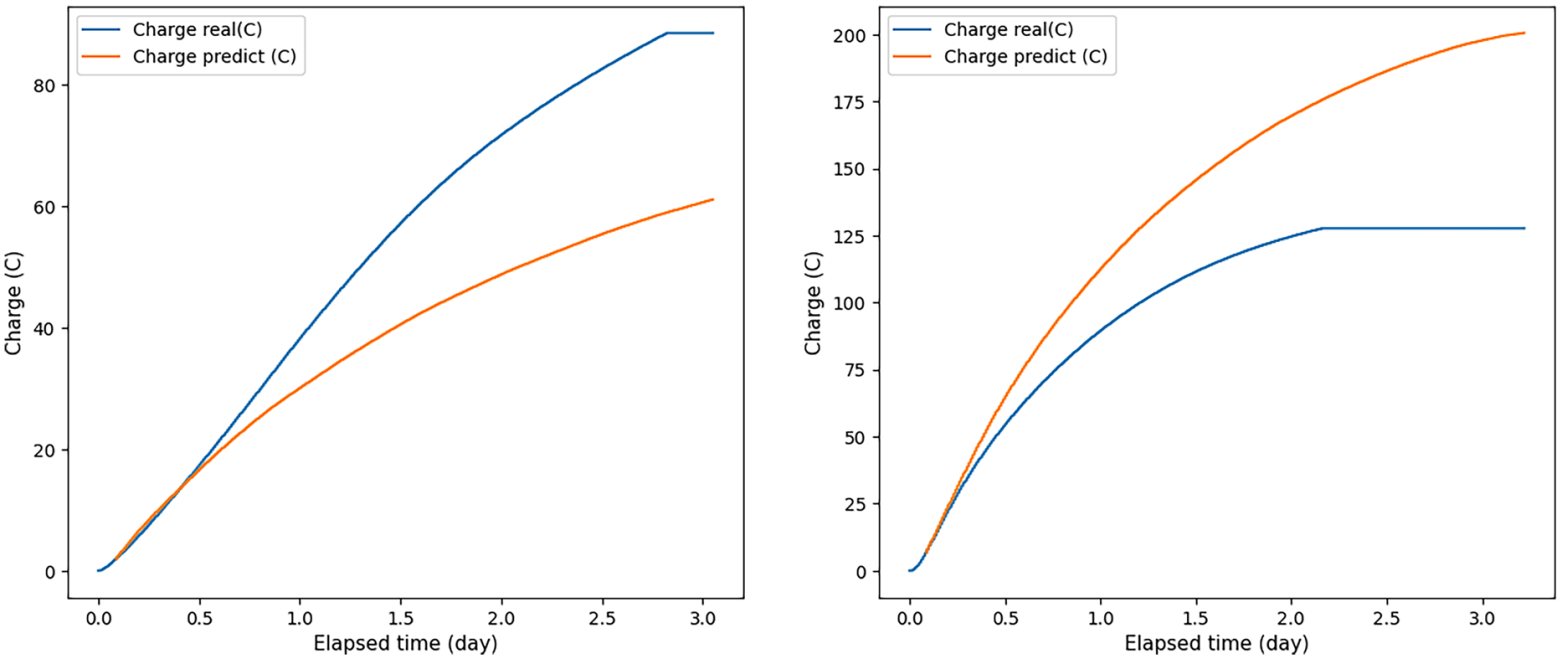
The ratio of the real and predicted charge, when voltage values measured over 2 h were applied to the ANN input. Blue graph—charge values obtained from real data; orange—charge values obtained from predicted data

**Fig. 9:**
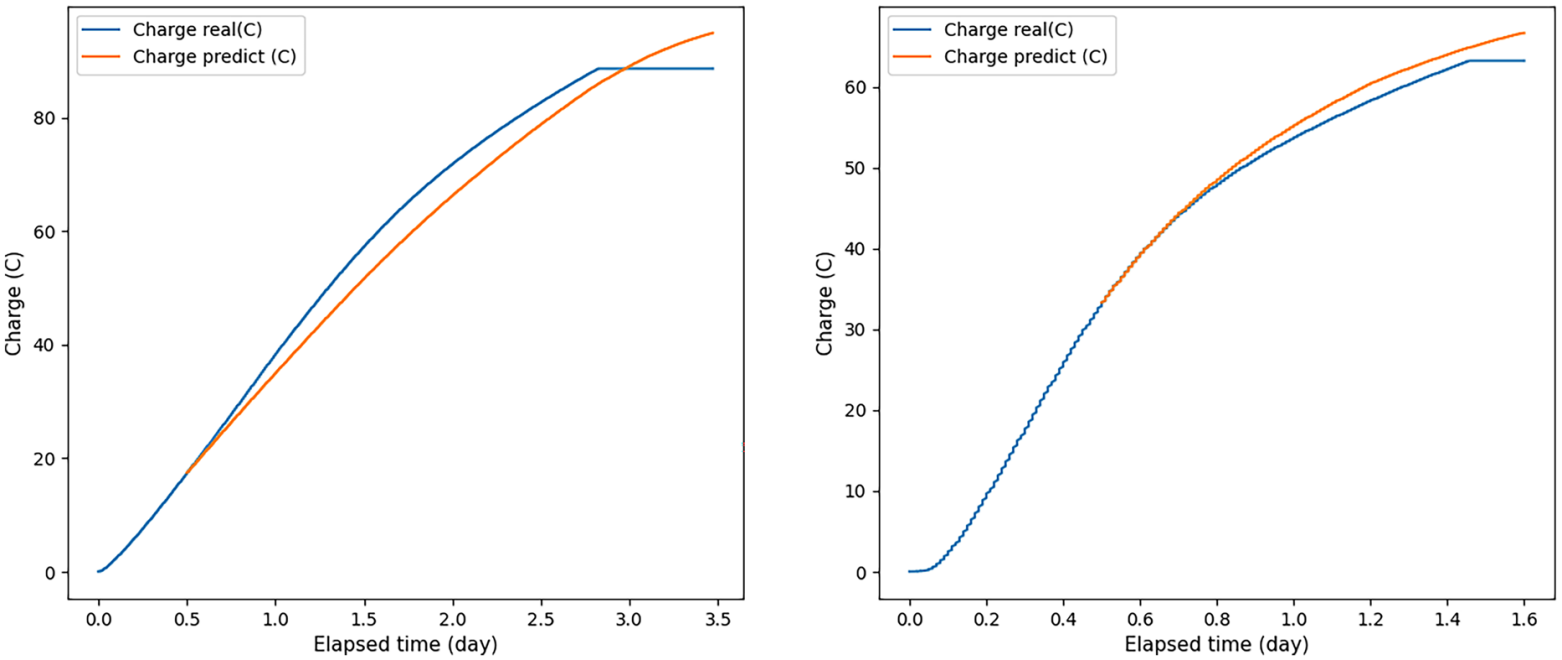
The ratio of the real and predicted charge, when voltage values measured over 12 h were applied to the ANN input. Blue graph—charge values obtained from real data, orange—charge values obtained from predicted data

Knowing the voltage and external resistance, the current strength was calculated according to Ohm’s law. Then, the total charge was calculated by numerically integrating the current as a function of time. At the same time, considering that during the development of the ANN, empty voltage values were filled with zeros up to 5 days, when calculating the integral, regression voltage values were discarded, which were less than 0.01 V. To equalize the dimensionality of all experiments, we filled in the missing stress values with zeros until day 5, so the regression stress gradually tended to zero over time, but these near-zero values could introduce an additional error in obtaining BOD_5_. At the same time, if we cut off the regression voltage at 0.02 V, we could finish the experiment earlier than the real one would go, since the regression voltage did not always reach 0.02 V exactly at the time when 0.02 V was achieved at a real experiment. Therefore, it was decided to cut off the regression voltage when it had already passed the cutoff point but had not yet reached zero, namely, when it was less than 0.01 V. Several examples of the ratios of the charge obtained from real data and the charge obtained from predicted data are shown in Figs. 8 and 9.

**Fig. 10:**
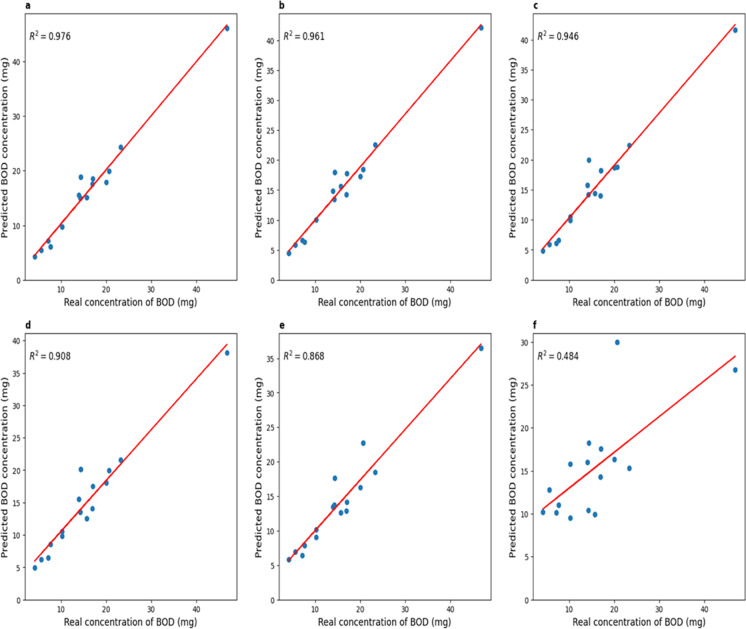
Plots comparing actual and predicted BOD_5_ values (for the indirect prediction approach). **a** When using 24-h voltage data; **b** for 16 h; **c** 12 h before; **d** 8 h; **e.** 6 h; **f** for 2 h

Due to the linear dependence of BOD_5_ and charge, the resulting total charge was used to estimate BOD_5_. Moreover, the values described in paragraph 3.1 (MAPE, MAX,] and *R*^2^) were used for the assessment. The results of these values for comparison BOD_5_ obtained by the formulas of linear dependence on the predicted charge with reference values BOD_5_ are shown in Table 5. Graphs of comparison of real and predicted BOD_5_ values for the case of indirect forecasting are shown in Fig. 10.

**Table 5:** Errors of BOD_5_ obtained from the predicted charge

Input	MAPE	MAX	*R*^2^
2 hours	48.35 %	20.06 mg	0.483
6 hours	15.62 %	10.32 mg	0.867
8 hours	11.95 %	8.72 mg	0.907
12 hours	10.66 %	5.53 mg	0.946
16 hours	8.42 %	4.69 mg	0.961
24 hours	7.5 %	4.49 mg	0.976

As in the case of direct prediction BOD_5_, sensitivity, specificity, and accuracy were calculated. The sensitivity was 1 when the input data were 24, 16, and 12 h of measurements, 0.9 for 8 h of measurements, and 0.8 for other cases (6, 2 h). Specificity was 1 for 24, 16, 12, and 8 h of measurements 0.5 for 2 h. The accuracy was 1 for the inputs at 24, 16, and 12 h of measurements, 0.9375 for 8 h, 0.875 for the input at 6 h, and 0.6875 for the input of 2 h of measurements.

### Discussion

As a first approach, ANN models were used to directly predict the values of BOD_5_, one value for each experiment. With this method, acceptable results were obtained when compared to reference values of BOD_5_ for 24, 16, and 12 h of measurement. This approach is more reliable since only one value is predicted, but it is less informative since it does not reflect the process of voltage change.

Using the second approach, ANN models predicted the voltage values from which a charge can be calculated and, consequently, BOD_5_. Moreover, as soon as the predicted voltage data reached small values (Tardy et al., 2021), a value of 0.02 V was determined as the end point of the measurement, and then the charge was considered until the day when the predicted voltage became less than 0.01 V. In comparison with the first approach, the results were better (see Tables 4 and 5), but for entries at 6 and 2 h, and in some experiments for entries at 8 and 12 h, the results, as in the first approach, gave a high error because voltage did not reach the peak and did not begin to decrease by this time.

As described in Tardy et al. (2021), the average error in the early detection of BOD_5_ using MFC did not exceed 10%. For example, for input data measured over 24 and 16 h, the average relative error did not exceed 10%, which corresponds to the error of the MFC in detecting BOD_5_ described in (Tardy et al., 2021).

## Conclusions

ANN models were trained on voltage data obtained by MFC for 24, 16, 12, 8, 6, and 2 h and used to predict BOD_5_ values. Two approaches were considered in the prediction of BOD_5_—when the ANN directly predicts BOD_5_, and when the ANN predicts voltage, from which BOD_5_ can be calculated. The results obtained during cross-validation and on the delayed test set did not differ much from each other. When using the voltage values obtained at 12 h as input, the error on the delayed set was 13.59% in the first approach and 10.66% in the second. For cases when voltage values measured for more than 12 h were input to the models, the relative error was even smaller, for an entry at 24 h, the relative error was 8.72% and 7.5% for the first and second approaches, respectively. ANN models for these cases showed good results regardless of the water sample used (domestic or brewery wastewater). Namely, the problem of determining the minimum measurement time required for a sufficiently accurate determination of the BOD_5_ was solved. Rapid acquisition of BOD_5_ values can offer benefits for wastewater monitoring and treatment. This will enable us to react faster, take necessary actions promptly, and identify optimal treatments under changing needs. It will also help to reduce costs and assist in being compliant with legal requirements to maintain BOD_5_ under certain levels.

For future work, more complex neural network methods will be explored. The goal will be to identify if it is possible to further improve the performance and reduce the number of monitoring hours. One such network is the transformer network used for time series forecasting (Li et al., 2019) which has shown strong results. It identifies local relationships from the given sequence, while also maintaining long-term memory dependencies. The transformer network can be adapted for both direct and indirect BOD_5_ predictions.

## Data Availability

The datasets generated and analyzed during the current study are available in the repository https://github.com/MedvedevIvanV/ANN-BOD5.
